# Secondary fracture prevention with osteoporosis medication after a fragility fracture in Sweden remains low despite new guidelines

**DOI:** 10.1007/s11657-023-01312-z

**Published:** 2023-07-29

**Authors:** Stina Ek, Anna C. Meyer, Maria Sääf, Margareta Hedström, Karin Modig

**Affiliations:** 1 Unit of Epidemiology, Institute of Environmental Medicine, Karolinska Institutet, Stockholm, Sweden; 2 Department of Molecular Medicine and Surgery, Karolinska Institutet, Stockholm, Sweden; 3 Department of Clinical Science, Intervention and Technology (CLINTEC), Karolinska Institutet, Stockholm, Sweden; 4 Trauma and Reparative Medicine Theme (TRM), Karolinska University Hospital, Stockholm, Sweden

**Keywords:** National registers, Older adults, Falls, Injuries

## Abstract

***Summary*:**

This national register study investigated osteoporosis medication prescriptions for secondary fracture prevention among older Swedish adults. Although prescription proportions are increasing for both men and women, they remain low, especially among individuals aged 90 and older. Improved Fracture Liaison Services could increase the prescription proportions and should be bolstered.

**Purpose:**

Despite clear guidelines for secondary fracture prevention among older adults, it seems undertreatment persists. We aimed to describe secondary fracture prevention with medical osteoporosis treatment in the older Swedish population. Specifically, how prescription has changed over time and if these patterns differ in sociodemographic subgroups.

**Methods:**

Between 2007 and 2020, osteoporosis medication use was studied among all Swedish residents aged 70 and older who had a fragility fracture in the previous 5 years. Previous fragility fractures were defined as fractures of the humerus, wrist, hip, or vertebrae. Osteoporosis medication was defined as any prescription of bone-enhancing medications (including bisphosphonates and denosumab).

**Results:**

Osteoporosis medication increased over the study period, especially among men. Prescription among individuals 90 + was consistently two- to threefold lower compared to 70–79- and 80–89-year-olds. In 2018–2020, 8–17% of women and 5–9% of men, depending on age, received osteoporosis medication. At the beginning of the study period, women with higher education were more likely to be prescribed osteoporosis medication, though this difference decreased over time. Prescription of vitamin D and/or calcium as the only treatment was more common than osteoporosis medications throughout the study period.

**Conclusion:**

Despite increasing prescription proportions, medical treatment for secondary fracture prevention remains low. In addition, it is more common to be prescribed vitamin D or calcium than osteoporosis medication after a fragility fracture, contrary to current guidelines. These results indicate that there is room for improvement for Fracture Liaison Services in Sweden.

**Supplementary Information:**

The online version contains supplementary material available at 10.1007/s11657-023-01312-z.

## Introduction

Osteoporosis is a public health problem and a major cause of disability-adjusted life years (DALYs) [1]. Fragility fractures in older adults are the most severe consequence of osteoporosis and affect one in two women and one in four men in Sweden and the burden is increasing in Europe, Asia, and the USA [2–5]. Fragility fractures are associated with severe costs for both patients and society [6]. To counteract suffering and costs, secondary prevention after a fragility fracture is recommended. Such pharmacological secondary fracture prevention has proven to be effective in slowing bone loss and decreasing the risk of new fractures [7]. The most common medications are bisphosphonates, shown to reduce vertebral fractures by 40–70% and hip fractures by 40–50% [8]. In Sweden, like in many other countries, there are national guidelines for how osteoporosis medication should be prescribed. These guidelines state that all postmenopausal women and all men over 50 years old who experience a fragility fracture should be considered for intervention with osteoporosis medication to prevent further fractures [9]. For more severe fractures (e.g., hip fracture and vertebrae fracture), medications could be prescribed without further evaluation. However, despite clear guidelines, undertreatment continues to be an issue [2, 10].

Estimated osteoporosis prevalence in Sweden for adults aged 50 and older was 22% among women and 7% among men [11]. However, due to a lack of screening and inconsistencies in bone mineral density (BMD) management programs, it is difficult to estimate the true prevalence of osteoporosis in the population and the observed proportion is likely underestimated. As an example, a Swedish study found that only 22% of older women eligible for osteoporosis treatment according to national guidelines received medical treatment [12]. IOF estimates the treatment gap (individuals with osteoporosis without medical treatment) to be 67% in 2021 [11]. The gap is likely greater among men than among women because osteoporosis is less often considered a diagnosis among men, even among those who have had a fracture, who are thus less often considered for medical treatment [13–15].

While several studies suggest undertreatment of osteoporosis after a fragility fracture, population-wide assessments are still scarce. Moreover, knowledge regarding if and how osteoporosis medication is prescribed to a similar extent in population subgroups is needed. If we can, for example, identify groups where the prescription proportion is lower than in other groups, preventive strategies can be directed. Therefore, with this study, we aimed to investigate and describe secondary fracture prevention with medical osteoporosis treatment among the older Swedish population. In the absence of a population measure for osteoporosis, we use a fragility fracture (fracture of the vertebrae, hip, wrist, or humerus) caused by low-energy trauma as a proxy, given that such fractures in older adults are most often associated with osteoporosis [16]. We specifically examine how prescription has changed over time and difference between men and women, age groups, and education level.

## Material and methods

### Study population

We identified all individuals in Sweden aged 70 years and older between 1 January 2007 and 31 December 2020 who had experienced a fragility fracture within the past 5 years. The population was identified through the Total Population Register (TPR) and fragility fractures were identified in the National Patient Register, which covers everyone that receives care in Sweden. Through the personal identification number (PIN) assigned to all Swedish residents, information from the registers could be linked at the individual level. Fragility fracture was defined as any fracture of the humerus (S42.2–S42.4), lower forearm (S52.5 and S52.6), hip (S72.0–S72.2), or vertebral (S12, S22.0, S22.1, S32.0, and T08) [17].

### Medications

In Sweden, all medications prescribed by licensed health care staff and retrieved from a pharmacy are registered at the individual level for all residents in the Prescribed Drugs Register (PDR) since 2006. Osteoporosis medications from PDR were recorded per calendar year and included the medications presented in Table 1. Bisphosphonates, strontium ranelate, denosumab, intact parathyroid hormone, and teriparatide were grouped and labeled “osteoporosis medications.” A timetable for when the specific osteoporosis medications were prescribed during the study period is shown in Appendix figure 1. In addition, vitamin D and/or calcium and systemic medium-strength estrogens and tibolone (further mentioned as “estrogens”) were also studied because these medications protect bone health to some extent and can be prescribed as supplements or an alternative to osteoporosis medication, despite current guidelines discouraging prescribing only calcium and vitamin D if no proven deficit. Medium-strength estrogens are neither recommended for prevention of fractures in older women.

**Table 1:** Included medications from the prescribed drug register

Type		ATC code
Vitamin D (cholecalciferol)/calcium		A12AA02, A12AA04, A12AA06, A12AX, A11CC05
Systemic medium-strength estrogens and tibolone		G03CA03 (except for vaginal), G03CX01, G03FA01, G03FA12, G03FA15, G03FA17, G03FB05, G03FB06, G03FB09
Osteoporosis medications	Teriparatide/intact parathyroid hormone	H05AA02, H05AA03
	Bisphosphonates, peroral and intravenous zoledronic acid	M05BA01, M05BA04, M05BA07, M05BA08, M05BB01, M05BB02, M05BB03, M05BB05
	Strontium ranelate	M05BX03
	Denosumab	M05BX04

In Sweden, in addition to the medications that are dispensed from pharmacies, there are medications bought directly from the manufacturer that are also dispensed in hospitals, primary care centers, and care homes. This system is called “medication per requisition” and these medications are given to patients in each facility without being registered in the PDR. Currently, no register of these drugs on an individual level exists; however, in relation to all medications prescribed, the requisitions constitute a minor part. Data on these medications can only be withdrawn on an aggregated level, such as defined daily dose (DDDs) for a period and region. This means that we can estimate how much of the medication of interest is being used, but not on an individual level.

### Covariates

Data on the sex and age of the individuals in the study was taken from the TPR. The highest education level for each individual was retrieved from the longitudinal integrated database for health insurance and labor market studies (LISA). Education was categorized into three groups: primary, secondary, and university education. Information about cohabitation was collected from LISA for the year prior to the fracture. Information about emigration was retrieved from the TPR and used for censoring individuals at the date of their first emigration. Date of death was retrieved from the Cause of Death Register.

### Statistical analysis

This study was conducted in several steps. First, we calculated the proportion of the fragility fracture population that had been prescribed osteoporosis medication. This was done separately for men and women and for different age and educational groups for each calendar year between 2007 and 2020. We estimated the relative odds of being prescribed osteoporosis medication within 1 year of the initial fracture in sex-stratified logistic regression models adjusted for age, education level, and cohabitation status. Individuals that died within 30 days from the fracture were excluded from the analysis because they most likely were not considered for medicine prescription. Finally, the change in the proportion of medication for osteoporosis and other types of bone health-related medications before and after a fragility fracture was visualized using flowcharts. This was done by taking information about medications 2 years prior to a fracture and 1 year after the fracture. We included osteoporosis medication and other bone health-related treatments, like vitamin D, calcium, and estrogen.

To complement individual-level prescribed data in the PDR, we calculated DDDs per calendar year for osteoporosis medications from the PDR and the requisition medications separately between 2011 and 2020. The requisition medication data is at the population level, so it cannot be directly compared to the individual-level data among older adults, but it provides information about general prescription trends.

SAS was used for data management and Stata and R Studio were used for statistical analyses.

## Results

In 2020, 8.5%, 15.3%, and 25.5% of women aged 70–79, 80–89, and 90 + had a fragility fracture. Among men, aged 70–79, 80–89, and 90 + , 3.4%, 7.0%, and 14.0% experienced a fragility fracture. Although the absolute number of fragility fractures has increased over the study period because of a growing and aging population, the proportions of persons with a fragility fracture have remained stable over time (see Appendix table 1). Table 2 shows the sociodemographic characteristics of the study population that had endured a fragility fracture in the last 5 years between the years 2007 and 2020.

**Table 2:** Characteristics of women and men with a previous fragility fracture during 2007–2020

	Women			Men		
	70–79 years	80–89 years	90 + years	70–79 years	80–89 years	90 + years
n	83,645	95,249	40,078	35,837	42,192	15,528
Living alone^**1**^	43,719 (52.3)	70,035 (73.5)	36,906 (92.1)	16,113 (45.0)	19,327 (45.8)	9416 (60.6)
Education						
Primary	32,130 (38.4)	54,315 (57.0)	26,728 (66.7)	14,748 (41.2)	21,669 (51.4)	8525 (54.9)
Secondary	32,831 (39.3)	29,141 (30.6)	10,079 (25.2)	13,811 (38.5)	13,682 (32.4)	4791 (30.9)
University	18,684 (22.3)	11,793 (12.4)	3271 (8.2)	7278 (20.3)	6841 (16.2)	2212 (14.2)

^1^At the beginning of the year of the fracture

### Time trends

**Fig. 1:**
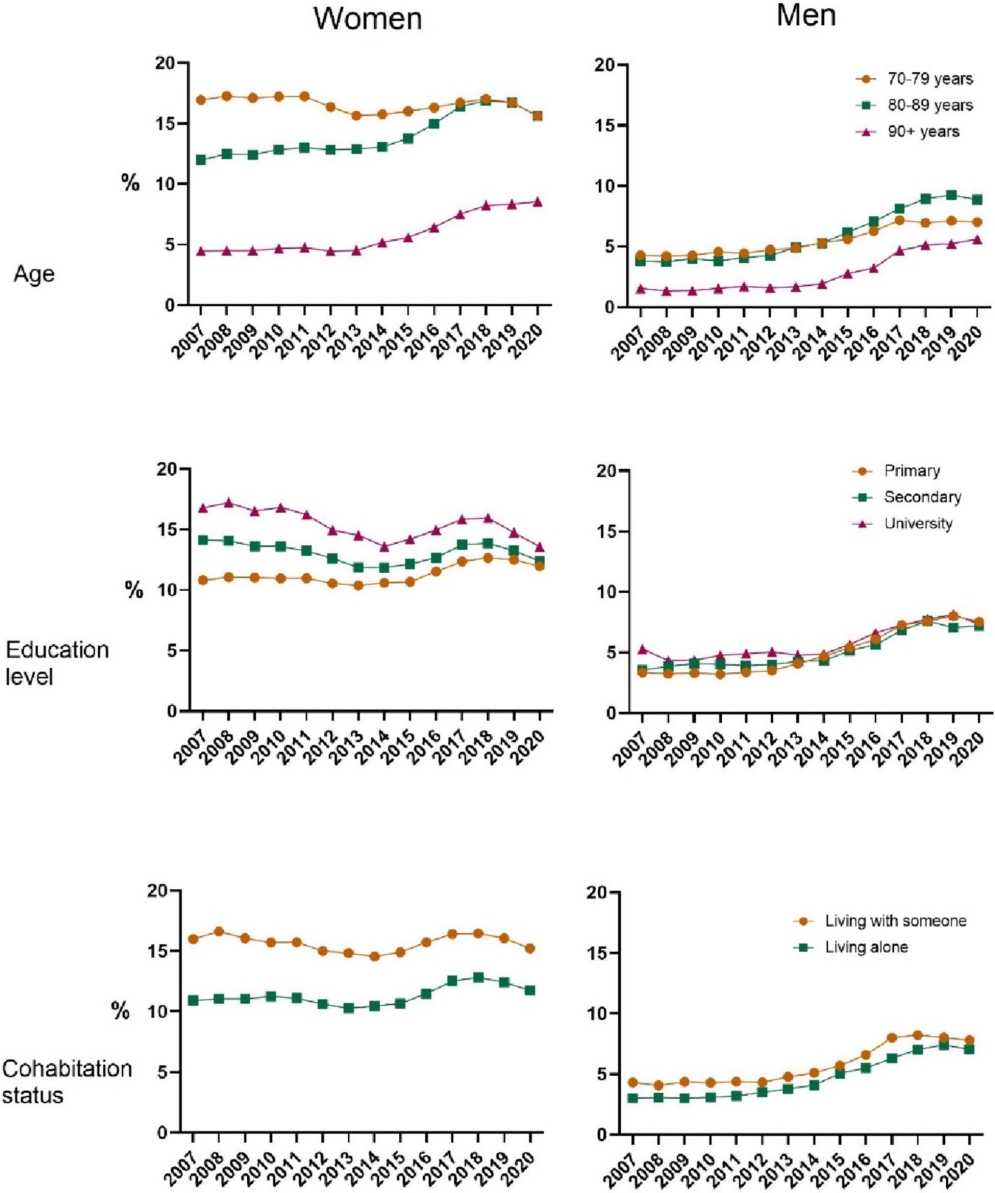
Time trends per age group in pharmacy-prescribed osteoporosis medication between 2007 and 2020, among women and men with a previous fragility fracture, presented by age group, education level, and cohabitation status

Figure 1 presents prescribed osteoporosis medication for women and men with a previous fragility fracture, stratified by age group, education level, and cohabitation status over the period 2007 to 2020.

In 2020, prescription in the oldest age group (90 +) was 8.6% among women and 5.6% among men, which was consistently two- to threefold lower compared to the other age groups. Over all age groups, prescriptions increased over time among both women and men. However, comparing women aged 70–79 to women aged 80–89, although prescription proportion was 15.6% in 2020 for both age groups, this represented a decrease in the 70–79-year-olds and an increase in the 80–89-year-olds. Among men, the proportion prescribed osteoporosis medication increased for all age groups, yet prescriptions were lower in all age groups compared to women. To wit, 2018–2020, 8–17% of women with a prior fragility fracture received osteoporosis medication while only 5–9% of men did. The education-stratified time trends show that at the beginning of the study period, the prescription proportion was highest among higher educated women. However, by the end of the period, the differences were no longer present. Women who live alone have a lower proportion of prescribed osteoporosis medications; however, this difference is partly driven by age, because older women are more likely to live by themselves but are less likely to be prescribed osteoporosis medication. Among men, prescription proportion is similar across all education groups and only slightly lower among those living alone.

### Medications per requisition

The complementing analyses on aggregated data for osteoporosis medication per requisition in the whole Swedish population revealed that medication per requisition was rare at the start of the study period but has increased. Since 2011, an increasing proportion of all osteoporosis medication is given in hospitals, primary care centers, and care homes compared to through prescriptions. The main part of the requisition medications is denosumab (Appendix figure 2). It should also be noted that the medication per requisition is based on the total Swedish population and potentially for other, mainly oncological indications such as hypercalcemia and bone metastases, while the medications from pharmacies are prescribed for an older sub-population with fragility fractures.

### Bone health-related medications before and after a fragility fracture

Table 3 presents the results of the logistic regression models investigating the association between fracture and odds of being prescribed osteoporosis medication accounting for age, living alone, and education. The highest odds of being prescribed osteoporotic medication was in the age group 70–79. However, as seen in Fig. 1, the difference between the age groups 70–79 and 80–89 diminishes over the study period. Cohabitation is associated with a slight increased odds of being prescribed osteoporotic treatment after a fragility fracture compared to those living alone among women (OR 1.16, (95% CI 1.13–1.19) and men (OR 1.17, 95% CI 1.11–1.24). Secondary and university education is associated with increased odds of prescription compared to primary education, but only among women (OR 1.10, 95% CI 1.07–1.14 and OR 1.18, 95% CI 1.14–1.22), and this difference decreased over time (Fig. 1).

**Table 3:** Multivariable odds ratios (95% confidence interval) for age group, cohabitation status, and education level and being prescribed osteoporosis medication within 1 year from a fragility fracture among women and men 2007–2020

	Women *n* = 210,113	Men *n* = 84,219
Age, in years		
70–79	Ref	Ref
80–89	**0.75 (0.73–0.77)**	**0.94 (0.88–0.99)**
90 +	**0.29 (0.28–0.30)**	**0.46 (0.41–0.51)**
Living alone	Ref	Ref
Living with someone	**1.16 (1.13–1.19)**	**1.17 (1.11–1.24)**
Education		
Primary	Ref	Ref
Secondary	**1.10 (1.07–1.14)**	1.05 (0.99–1.12)
University	**1.18 (1.14–1.22)**	1.07 (0.99–1.15)

Bold text = statistically significant

**Fig. 2:**
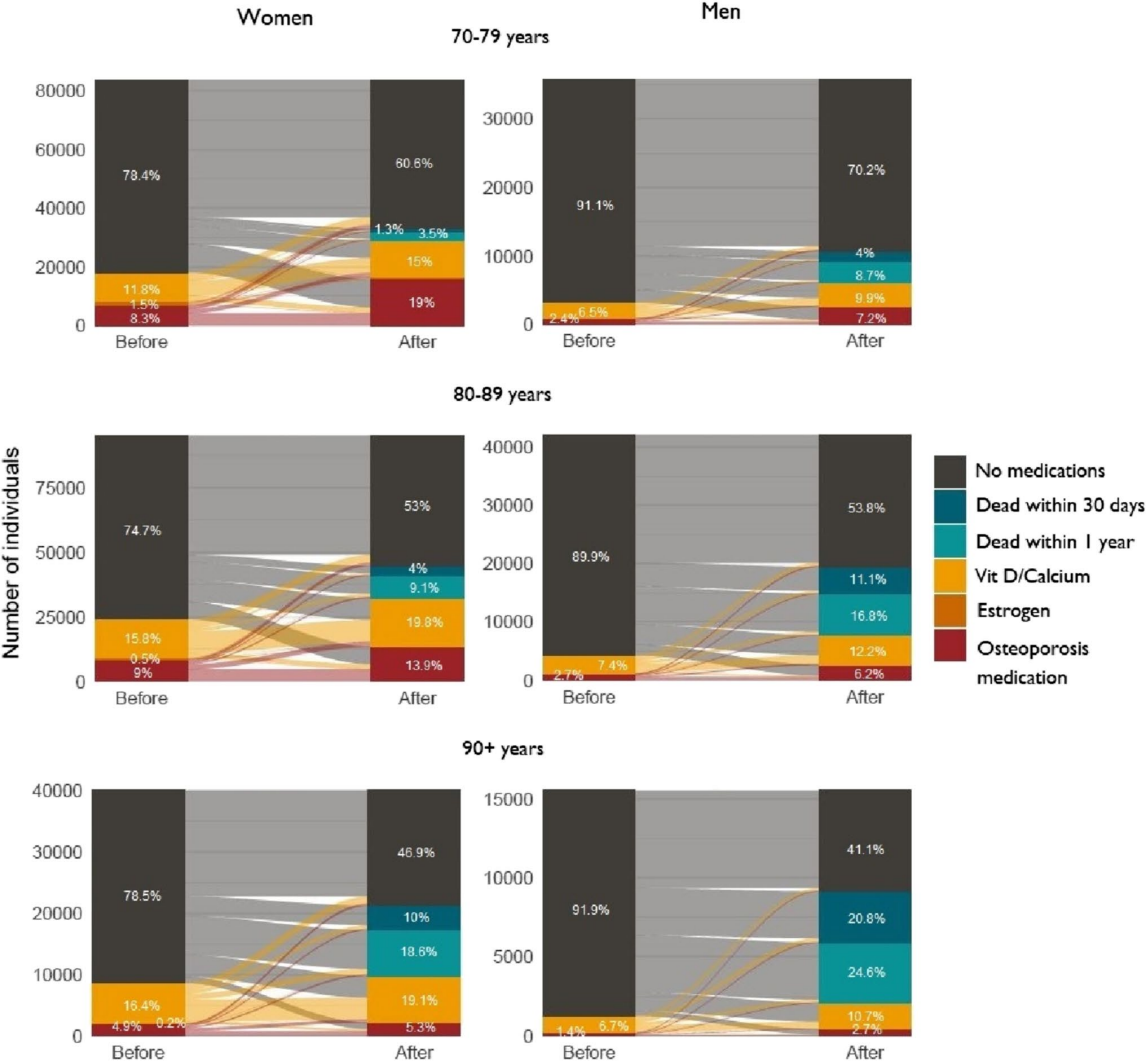
Proportion of individuals with a previous fragility fracture that during 2007–2020 were prescribed osteoporosis medication, vitamin D/calcium, or estrogen before and after the fracture. Stratified by sex and age groups. (“Osteoporosis medication” could be in combination with medium strength estrogens or vitamin D/calcium, “Estrogens” either only estrogens or in combination with vitamin D/calcium, and “Vitamin D/Calcium” is only vitamin D and/or calcium)

The visualization of prescriptions of osteoporosis medications, vitamin D, calcium, and estrogen before and after a fracture is shown in Fig. 2. The flowchart shows that most individuals, between 75 and 92%, depending on age and sex, did not have any type of bone-strengthening medication prior to their fragility fracture. Vitamin D and/or calcium (7–16%) was the most prescribed treatments and was even more common after a fracture (11–20%). Prior to a fracture, osteoporosis medications were prescribed to only 1–9% of individuals, depending on age and sex. After a fracture, the proportion increased to 10–19% when also considering those who had a short survival. We created the same flowcharts for the period before and after the implementation of new guidelines in 2015 (Appendix figure 4); these show a slight increase in osteoporosis medication prescription after the new guidelines but still low proportions overall with vitamin D/calcium accounting for largest proportion of prescriptions.

## Discussion

The aim of this study was to investigate and describe how pharmacological osteoporosis treatment is prescribed following a fragility fracture in the older Swedish population. We used high-quality register data from all Swedish residents aged 70 years and older who had experienced a fragility fracture, with information about all their prescriptions extracted from pharmacies. Our results show that even though prescriptions of osteoporosis medications have increased over time, it is still at rather low levels in a population with a clear indication for treatment of osteoporosis. The proportion of men with osteoporosis prescriptions is especially low although doubled between 2007 and 2020. For women, the increase over time is more modest. The proportion of people with higher education with osteoporosis prescriptions was higher than the proportion of people with lower education; this difference was greatest at the beginning of the study period, but this gap has closed in recent years. A difference in prescription proportion between high- and lower-educated women has also been shown by Wastesson et al. who investigated differences in osteoporosis medication between educational levels with data from 2005 [18]. The slight decrease in medication proportion for highly educated women in the most recent years is surprising but might be due to changes in the population composition over the study period (e.g., higher education became more common during the study period).

When additionally considering other medications with known bone positive agents (although limited effect), such as vitamin D, calcium, and estrogen, it was clear that supplementation with only vitamin D and/or calcium is the most common bone health-related treatment both before and after a fragility fracture. The current Swedish guidelines state that vitamin D and calcium can be given as a complement to osteoporosis medication, if an insufficiency exists, but not as the only treatment for osteoporosis. Our results show that it is still more common to get only vitamin D and calcium after a fragility fracture than to receive the recommended specific osteoporosis treatment, contrary to current guidelines.

The reasons for the low prescription proportions are likely several. Undertreatment due to lack of knowledge about the balance between side effects and benefit for the oldest frail population is one possible explanation. Individuals 90 years old and older have the lowest proportions of prescriptions, which is both expected and worrying. Some regions in Sweden advise against prescribing osteoporosis medication to very frail older adults that are not expected to live long enough to see the benefit of it. Meanwhile, we know that it is now more common to live an active life at older ages, and for these individuals, the stress that an osteoporotic fracture imposes might be a tipping point into frailty or an accelerated declining health and quality of life. In addition, the risk of recurrent fractures is high, which increases the cost for society and the suffering for the individual even more [19, 20]. It is therefore important to consider not only a patient’s chronological age but also health status when deciding their medical osteoporosis treatment. Medical contraindications to prescribe, such as renal insufficiency, can be one reason contributing to the low prescription proportions [21]. There is a risk that clinicians avoid prescribing to the oldest old due to a sparsity of studies in this group, but also believe that the risks outweigh the benefits for vulnerable individuals. A study performed in Swedish health care has shown that Fracture Liaison Services (FLS) regimes, as a part of post-fracture care, could decrease the treatment gap for osteoporosis [22], with 32% of fracture patients getting treatment after a FLS implementation compared to 13% prior to FLS. FLS implemented in two Swedish hospitals also reduced recurrent fracture risk with 18% [23]. However, although the awareness and use of such programs are increasing, there is still a need to implement and cohere to FLS regimes [24]. Both studies were, however, small samples conducted in only one or two centers. Kanis et al. report that 25% of all hospitals in Sweden report to be using/have established FLS, to be compared with the UK and the Netherlands that have a 50% coverage of hospitals with FLS [25]. The same report concludes that the Swedish national guidelines are clear and of high quality. However, different specialists can be responsible for the FLS in different regions and hospitals, and several different types of FLS exist [25]. Sweden has special geographic and socio-economic conditions that can affect regional differences, the northern parts of the country are sparsely populated while the southern parts more densely populated, and the physician/habitant rate is also lower in the northern parts. In addition, health care is organized within politically governed regional organizations which strive to promote and strengthen local self-government. Despite this, the national guidelines about evaluation and treatment from the Department of Health and Welfare and the Medical Products Agency are supposed to be implemented on a national level. These regional differences might partly explain the low prescription proportion among individuals with a previous fragility fracture. Our results indicate that improving the coordination of FLS regimes in Sweden, according to existing national guidelines, should be a prioritized action. Although regional differences in Sweden were outside the scope of this study, mapping regional differences to be able to lift regions that do not use FLS should be prioritized and a subject of future studies.

Another possible reason for the overall low prescription proportion is underdiagnosis. Despite clear guidelines, there seems to be a low frequency of screening among older fracture patients due to unclear BMD testing regimes and a lack of DXA scan machines in some parts of Sweden [26]. Targeted screening is recommended while general screening is not. However, the most recent guidelines state that medical treatment should be prescribed without further investigation for hip and vertebrae low-energy fractures [9, 27]. We show that osteoporosis in men with a fragility fracture seems to be even more undertreated than that in women, in line with previous studies [13, 14]. This might be because osteoporosis is typically considered a women’s disease and men with fragility fractures might not be evaluated to the same extent as women. According to Vescini et al., men experience fractures about 10 years later than women do, which might imply that they have more comorbidities and higher frailty levels, and thus worse outcomes after the fracture than women [13]. This could also impact the chance of being prescribed osteoporotic medication. Studies have shown that FLS are effective to mitigate inequity in osteoporosis medication, benefitting men and the oldest old [28, 29].

Our findings suggest that medical treatment after fragility fractures in Sweden does not adhere to recommendations and national guidelines. However, to clarify the level of undertreatment, we would need more detailed information on drugs prescribed within health care, per requisition. Our analyses on aggregated requisition data show that it is becoming more common in Sweden to distribute some drugs as requisition, among them injections of bisphosphonates or denosumab. However, comparison with the aggregated data should be made with caution because these numbers are based on all Swedes regardless of prior fracture or age. Moreover, some of the medications might be prescribed for other indications, such as treating hypercalcemia in cancer patients [30]. This means that the proportion shown in the appendix material is an overestimation with regard to osteoporosis medication administered through requisition. Denmark has a similar system as Sweden in terms of medications per requisition and a recent study has shown a pattern similar to our findings [31]. This might indicate that the increase in medications per requisition could explain the plateau effect that we see in prescribed medications in the recent years. It is also possible that part of the prescriptions of only vitamin D and calcium might be accompanied by osteoporosis medications on requisition, in accordance with guidelines. However, even when including the proportion of medications given on requisition, the total medical treatment of osteoporosis still appears low.

### Strengths and limitations

A strength in this study is the nationwide coverage of all prescribed medications picked up at pharmacies in Sweden. This limits the risk of selection bias. However, the medications included in our study have different treatment periods and frequencies; it can range from daily oral intake dispensed for several years to annual infusions. This makes it difficult to know which exact time window is optimal to capture prescriptions after a fracture. We decided not to distinguish first-time prescriptions from any prescription per calendar year to avoid misclassification of treatment. However, by allowing a time window of possible treatment after a fragility fracture for between 0 and 5 years, we tried to capture medical treatment that was a consequence of the fracture. This also helped to lower the risk of missing any prescribed medication that might have been delayed.

## Conclusion

Even if prescribed osteoporosis medications among fragility fracture patients have increased in the last decades, undertreatment persists despite clear national guidelines. Younger age groups, women, and people cohabiting with someone else have higher odds of osteoporosis prescription after a fragility fracture throughout the study period. Education level was only marginally associated with prescriptions, and only among women. Vitamin D and calcium are more commonly prescribed than specific osteoporosis medications, contrary to what the current guidelines recommend.

### Supplementary Information

Below is the link to the electronic supplementary material.

Supplementary file1 (DOCX 334 KB)

## Data Availability

Not available.

## Code availability

On reasonable request.
