# Supplementary material for: Secondary fracture prevention with osteoporosis medication after a fragility fracture in Sweden remains low despite new guidelines
Source: Arch Osteoporos. 2023 Jul 29;18(1):107. doi: 10.1007/s11657-023-01312-z (PMC10386957; doi:10.1007/s11657-023-01312-z)
Supplement: Supplementary file 1 — Supplementary file1 (DOCX 334 KB) [file 11657_2023_1312_MOESM1_ESM.docx]

Appendix material

**Appendix Table 1.** Number of individuals in the total population per age group and sex strata and proportion of individuals with a previous fragility fracture (%) per year, calculated 5 years previously.

|  | **Women** | | | **Men** | | |
| --- | --- | --- | --- | --- | --- | --- |
|  | *70-79 years* | *80-89 years* | *90+ years* | *70-79 years* | *80-89 years* | *90+ years* |
| **2007** | | | | | | |
| Total population | 364,704 | 273,761 | 70,138 | 309,716 | 174,329 | 26,921 |
| Fragility fracture | 9.2% | 16.6% | 26.2% | 3.4% | 7.3% | 14.3% |
| **2008** | | | | | | |
| Total population | 365,483 | 273,171 | 70,698 | 314,288 | 175,515 | 27,532 |
| Fragility fracture | 9.3% | 16.7% | 26.1% | 3.5% | 7.4% | 14.2% |
| **2009** | | | | | | |
| Total population | 369,362 | 271,224 | 71,485 | 321,435 | 175,399 | 28,513 |
| Fragility fracture | 9.3% | 16.8% | 26.0% | 3.5% | 7.5% | 14.2% |
| **2010** | | | | | | |
| Total population | 371,724 | 267,385 | 75,985 | 327,908 | 174,872 | 30,833 |
| Fragility fracture | 9.2% | 17.0% | 25.9% | 3.5% | 7.7% | 14.1% |
| **2011** | | | | | | |
| Total population | 376,808 | 263,742 | 79,109 | 336,583 | 174,360 | 32,684 |
| Fragility fracture | 9.1% | 17.0% | 26.2% | 3.5% | 7.7% | 14.3% |
| **2012** | | | | | | |
| Total population | 387,510 | 261,653 | 80,460 | 350,746 | 175,135 | 33,758 |
| Fragility fracture | 9.0% | 16.8% | 26.0% | 3.5% | 7.6% | 14.4% |
| **2013** | | | | | | |
| Total population | 403,920 | 258,064 | 81,146 | 369,476 | 174,522 | 34,719 |
| Fragility fracture | 9.0% | 16.6% | 25.8% | 3.5% | 7.5% | 14.6% |
| **2014** | | | | | | |
| Total population | 423,248 | 255,546 | 82,007 | 391,577 | 175,216 | 35,364 |
| Fragility fracture | 8.8% | 16.3% | 25.5% | 3.4% | 7.3% | 14.4% |
| **2015** | | | | | | |
| Total population | 443,347 | 253,892 | 83,263 | 412,472 | 177,440 | 36,404 |
| Fragility fracture | 8.8% | 16.2% | 25.6% | 3.4% | 7.3% | 14.3% |
| **2016** | | | | | | |
| Total population | 461,984 | 254,165 | 83,645 | 432,061 | 180,469 | 36,944 |
| Fragility fracture | 8.7% | 15.8% | 25.3% | 3.4% | 7.1% | 14.3% |
| **2017** | | | | | | |
| Total population | 479,252 | 255,073 | 83,839 | 449,163 | 184,798 | 37,331 |
| Fragility fracture | 8.6% | 15.5% | 25.1% | 3.4% | 7.1% | 14.0% |
| **2018** | | | | | | |
| Total population | 493,871 | 257,498 | 84,015 | 463,926 | 189,965 | 37,722 |
| Fragility fracture | 8.6% | 15.2% | 24.9% | 3.4% | 6.9% | 14.3% |
| **2019** | | | | | | |
| Total population | 505,076 | 261,818 | 83,760 | 474,901 | 196,584 | 38,101 |
| Fragility fracture | 8.6% | 15.0% | 24.8% | 3.4% | 6.8% | 14.1% |
| **2020** | | | | | | |
| Total population | 514,502 | 230,305 | 85,107 | 483,884 | 172,512 | 39,193 |
| Fragility fracture | 8.5% | 15.3% | 24.5% | 3.4% | 7.0% | 14.0% |


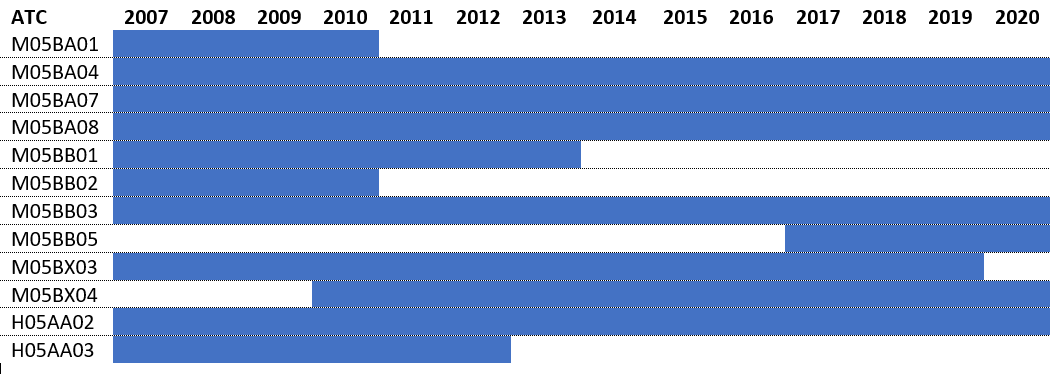


**Appendix figure 1.** Time period for any prescription per year of each type of medications, by ATC code.


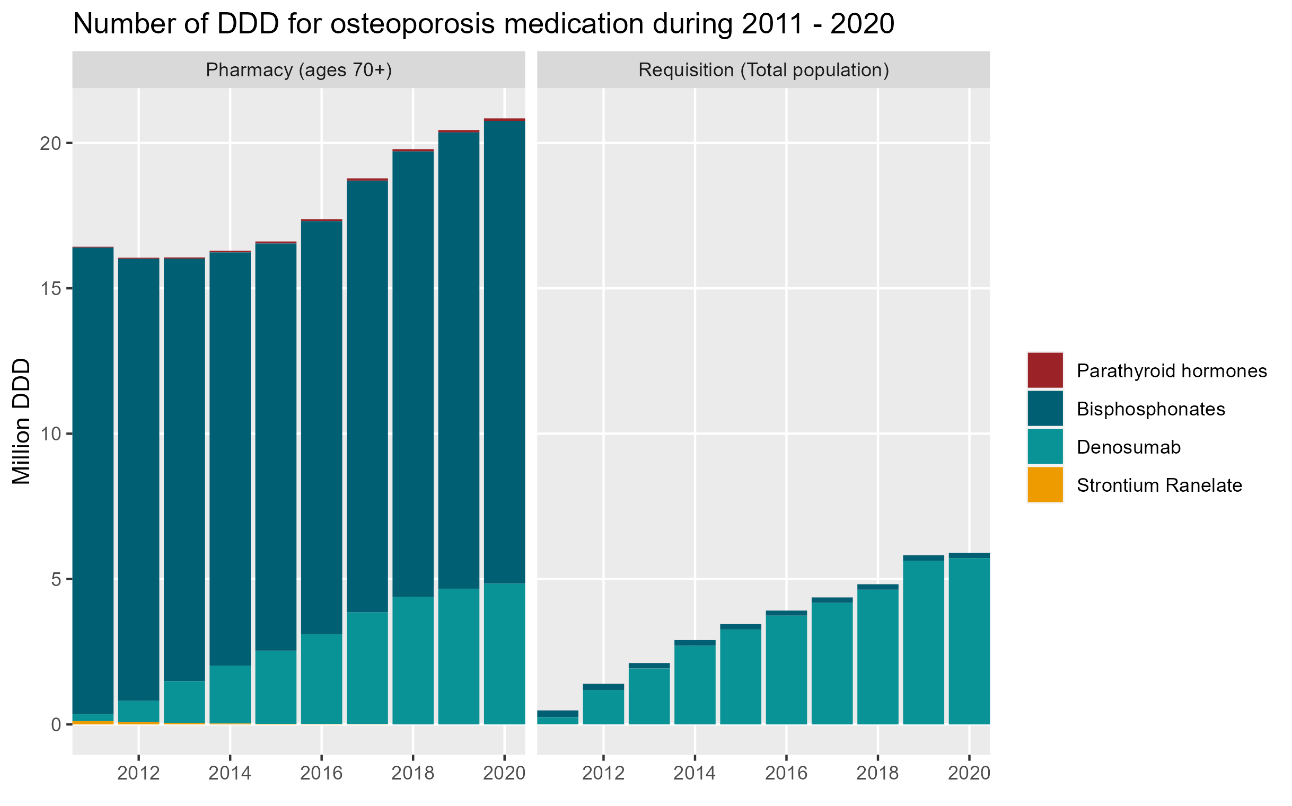


**Appendix figure 2**. DDD* of osteoporosis medication that is being prescribed in total for the whole population, from pharmacies and via requisition between 2011 and 2020. Note: included medications can also be prescribed for other conditions, such as hypercalcemia. *according to WHO/Swedish Medical Products Agency


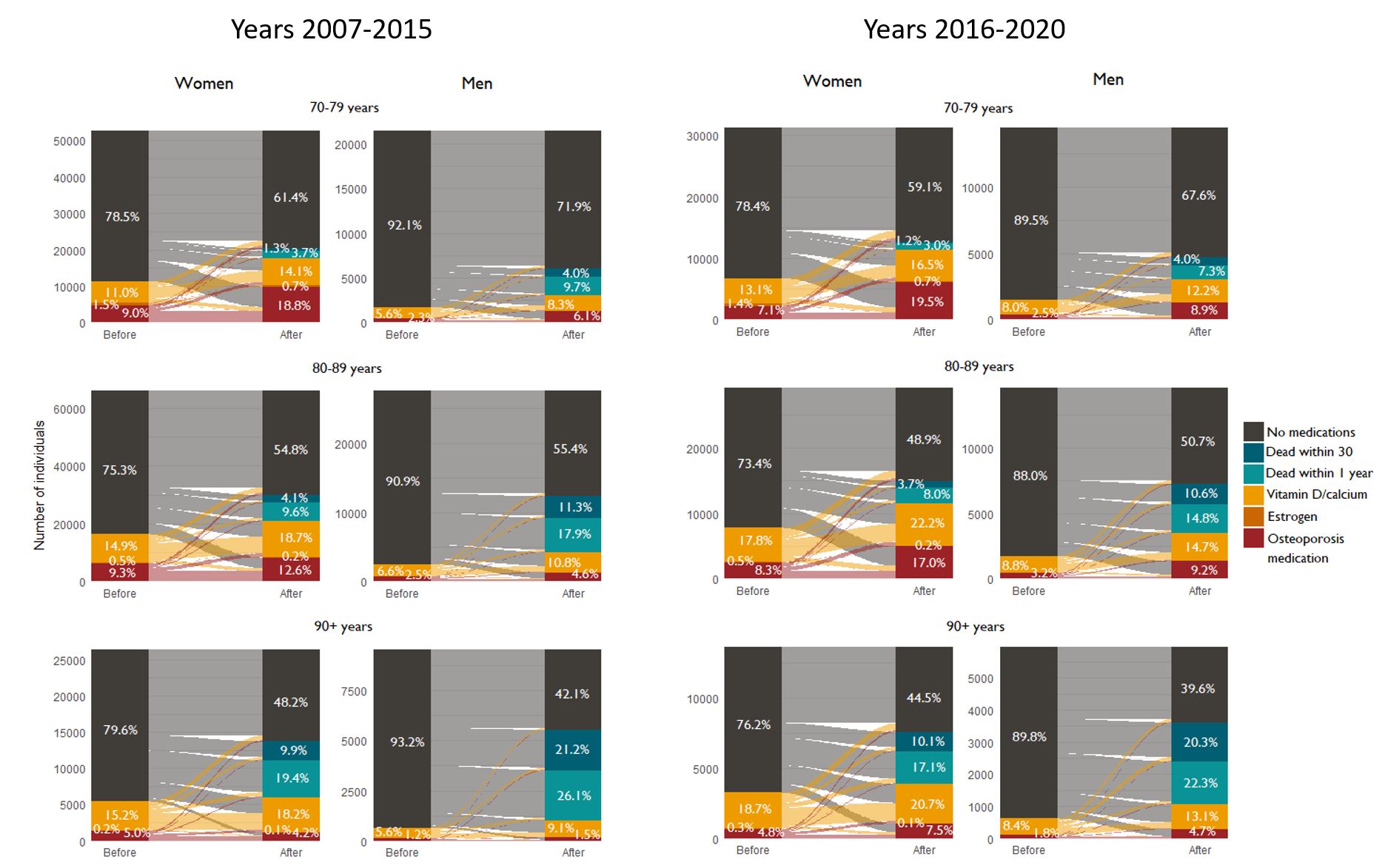


**Appendix figure 4**. Proportion of individuals with a previous fragility fracture that were prescribed osteoporosis medication, vitamin D/calcium, or estrogen before and after the fracture. Stratified by sex and age groups and shown for the years 2007-2015 and 2016-2020, reflecting new guideline implementation in 2015.
